# Energy Reallocation to Breeding Performance through Improved Nest Building in Laboratory Mice

**DOI:** 10.1371/journal.pone.0074153

**Published:** 2013-09-11

**Authors:** Brianna N. Gaskill, Kathleen R. Pritchett-Corning, Christopher J. Gordon, Edmond A. Pajor, Jeffrey R. Lucas, Jerry K. Davis, Joseph P. Garner

**Affiliations:** 1 Department of Animal Science, Purdue University, West Lafayette, Indiana, United States of America; 2 Charles River, Wilmington, Massachusetts, United States of America; 3 Environmental Protection Agency, Research Triangle Park, North Carolina, United States of America; 4 Department of Production Animal Health, University of Calgary, Calgary, Alberta, Canada; 5 Department of Biological Sciences, Purdue University, West Lafayette, Indiana, United States of America; 6 Department of Comparative Pathobiology, Purdue University, West Lafayette, Indiana, United States of America; 7 Department of Comparative Medicine and the Department of Psychiatry and Behavioral Sciences, Stanford University, Stanford, California, United States of America; Tulane University Medical School, United States of America

## Abstract

Mice are housed at temperatures (20-26°C) that increase their basal metabolic rates and impose high energy demands to maintain core temperatures. Therefore, energy must be reallocated from other biological processes to increase heat production to offset heat loss. Supplying laboratory mice with nesting material may provide sufficient insulation to reduce heat loss and improve both feed conversion and breeding performance. Naïve C57BL/6, BALB/c, and CD-1breeding pairs were provided with bedding alone, or bedding supplemented with either 8g of Enviro-Dri, 8g of Nestlets, for 6 months. Mice provided with either nesting material built more dome-like nests than controls. Nesting material improved feed efficiency per pup weaned as well as pup weaning weight. The breeding index (pups weaned/dam/week) was higher when either nesting material was provided. Thus, the sparing of energy for thermoregulation of mice given additional nesting material may have been responsible for the improved breeding and growth of offspring.

## Introduction

Typical housing temperatures of 20-24 ^°^C [1–3] are mild compared to conditions wild mice might encounter in the extremes of their natural habitats. In the laboratory, mice are able to live and reproduce without insulation from nests. These temperatures, however, are lower than the mouse’s lower critical temperature, a point at which the mouse’s metabolism begins to increase to counter heat loss, approximately 30^°^C [1]. Raising ambient temperatures in animal rooms to offset this disparity is not a solution as mice prefer different temperatures at different times of day, for different behaviors [4,5], and at different life stages [6,7]. Furthermore, increases in ambient temperature, even within the recommended range of 20-26 ^°^C, can increase aggressive interactions [8].

Mice in laboratory conditions must therefore use additional energy to stay warm [9]. To meet these increased thermoregulation-induced energy requirements, energy may be reallocated from other biological processes. The energetic costs of gestation and lactation are substantial for female mammals and when extreme thermal stress is imposed, reduced production and survival of offspring are seen [10,11]. Laboratory environments are cooler than 36-38^°^C, the temperature preferred by neonatal pups [6], and may potentially impact the well-being of young mice after they are born. At birth, mouse pups do not have the capacity to thermoregulate [12] and must rely on conductive and radiant heat from parents and other siblings in the nest and heat retained by the nest itself for survival [13,14]. After parturition, the most important factor for pup survival in the wild is the condition of the nest [15,16]; mice that build dome-shaped, rather than flat or cup-shaped, nests have better pup survival [11]. Similarly, laboratory mice selected for superior nest building have higher reproductive success than controls in both laboratory and extreme conditions [11].

Behavior is generally the animal’s first response to thermal stress and generally the most cost effective [1]. In the laboratory, mice provided with an appropriate amount [17] and type [18] of nesting material build better, more enclosed, nests. This improvement in nest building provides better insulation and reduced heat loss to the environment, resulting in reduced feed consumption [2,19]. This is consistent with a reduction in energy being burned for thermogenesis [2]. The provision of nesting material does not alter body core temperature or its variability [2], indicating that the mechanism of maintaining homeothermy has been shifted away from energetically expensive thermogenesis.

In this experiment, we hypothesized that suitable nesting material would allow mice to reduce heat loss, thus freeing up energy for reproduction and lactation, and subsequent pup growth. We predicted that mice with nesting material would build better nests than controls and that the increased insulation from nesting material, as was found in a previous experiment [2], would increase the number of pups born and weaned, decrease the amount of feed needed to produce those pups, decrease mortality (number weaned/ number born), and improve the breeding index (BI; pups weaned/dam/week). Furthermore, because insulated pups will stay warmer, once thermoregulatory processes have begun after approximately 10 days, pups born into a nest will use energy for growth rather than thermogenesis and will therefore be heavier at weaning.

## Materials and Methods

### Ethics statement

All experimental work involving live animals was approved by both the IACUC of Purdue University (Protocol 09-030) and the IACUC of Charles River (P03272009).

### Animals

All mice in this study were bred and housed at Charles River’s Association for the Assessment and Accreditation of Laboratory Animal Care, International accredited facility in Portage, MI. We housed naïve C57BL/6NCrl (C57BL/6), BALB/cAnNCrl (BALB/c), and Crl:CD1(ICR) (CD-1) breeding pairs (*n* = 30 pairs per strain/stock) in typical barrier rooms. The mice were bred in accordance with the standard breeding protocols of Charles River, in which breeding pairs that are not productive within 60 days after initial set up or 45 days after delivery of a first litter are euthanized.

### Housing

C57BL/6 and BALB/c mice were housed in one barrier room with an average temperature of 20.6^°^C and CD-1s were housed in a second room which averaged 20.1^°^C. Because our goal was to test nesting material in an existing commercial breeding setting, it was not possible to avoid confounding room with strain. Animals were housed in wire-topped polycarbonate shoebox cages (Lab Products, Inc., Seaford, DE), bedded with chipped hardwood bedding (Beta-Chip; NEPCO, Warrensburg, NY). Animals were kept on a 12:12 light: dark cycle (lights on at 05:00) and had *ad libitum* access to water and feed (Lab Diet 5L79; Purina Mills, Richmond, IN). Feed was weighed as added and weighed back at the end of the experiment to obtain total food use over the 6 month period. Colony animals within the barrier rooms were routinely monitored for infectious agents. All animals included in the study tested negative for a comprehensive list of viral, bacterial, fungal, and parasitic agents. Details may be found here: http://www.criver.com/SiteCollectionDocuments/hmsummary.pdf All testing was conducted Charles River’s Research Animal Diagnostic Laboratory; in Wilmington MA.

### Nesting material

Breeding pairs of one outbred and two inbred mice were housed with one of three nesting material treatments for 6 months (i.e. a 3x3 factorial design; n = 10 pairs per strain and treatment combination; a total of 90 breeding cages). Mice were provided either 8g of Enviro-Dri® (Fibercore, Cleveland, OH), 8g Nestlets® (Ancare, Bellmore, NY), or no nesting material (controls) at cage change each week. These treatments were chosen as the nesting treatments because they are commonly used materials and have been previously studied by our laboratory [2,5,17,18].

Nests were scored once a week using a 1-5 scale from a previously published protocol [18]. A score of 1: was manipulated material but no central nest cite was evident; 2: was a flat nest; 3: was a cup nest; 4: was an incomplete dome; 5: was a complete and enclosed dome (see [18] for further description of the scoring protocol). Nest scores were recorded from all treatments because mice will attempt to build a simple nest out of bedding material when other substrate is not provided [18].

### Breeding performance

Cages were observed three times per week and the number of pups born dead or alive and the number of pups weaned in each cage was recorded. Age at weaning (ranging between 16–32 days in this study) was subject to protocols within the barrier room. Pups were weighed and sexed at the time of weaning. We calculated an overall breeding index (BI) for each cage at the end of the experiment as the number of pups weaned per dam per week. The number of litters born per cage varied from 0-8 during the the 6-month study. Litters born before the end of the 6-month study were followed until weaning to record weaning weights and sex.

### Statistical analyses

Analyses were performed GLM in JMP 6 for Windows. All data, unless otherwise stated, were averaged per cage and were analyses using a simple model of Strain, Treatment, and their interaction. If the interaction was not significant, it was removed from the analysis and rerun without it. This means that if a strain-by-treatment interaction is not reported for an analysis then the reported treatment effect did not differ significantly between the strains. Logged average weaning weight per litter utilized a similar model as above but was blocked by cage and included age at weaning as a covariate and all second order interactions. The food consumption model also included the total number of pups weaned and the number of days caged as a covariate, since some mice were retired early due to being unproductive. Pup mortality was calculated per cage as 1-(number of pups weaned/number of mice born, either dead or alive). After data were collected, it appeared that nest scores declined when litters were born; therefore an additional term of Litter Presence was added to the nest score analysis to account for this difference. The assumptions of GLM (normality of error, homogeneity of variance, and linearity) were confirmed *post-hoc*, and appropriate transformations were made to meet these assumptions [20]. Significant effects were then analysed using *post-hoc* Tukey tests or Bonferroni corrected planned contrasts using custom contrasts in JMP. All values are given as least squares means and standard error.

## Results

### Nest Scores

**Figure 1:**
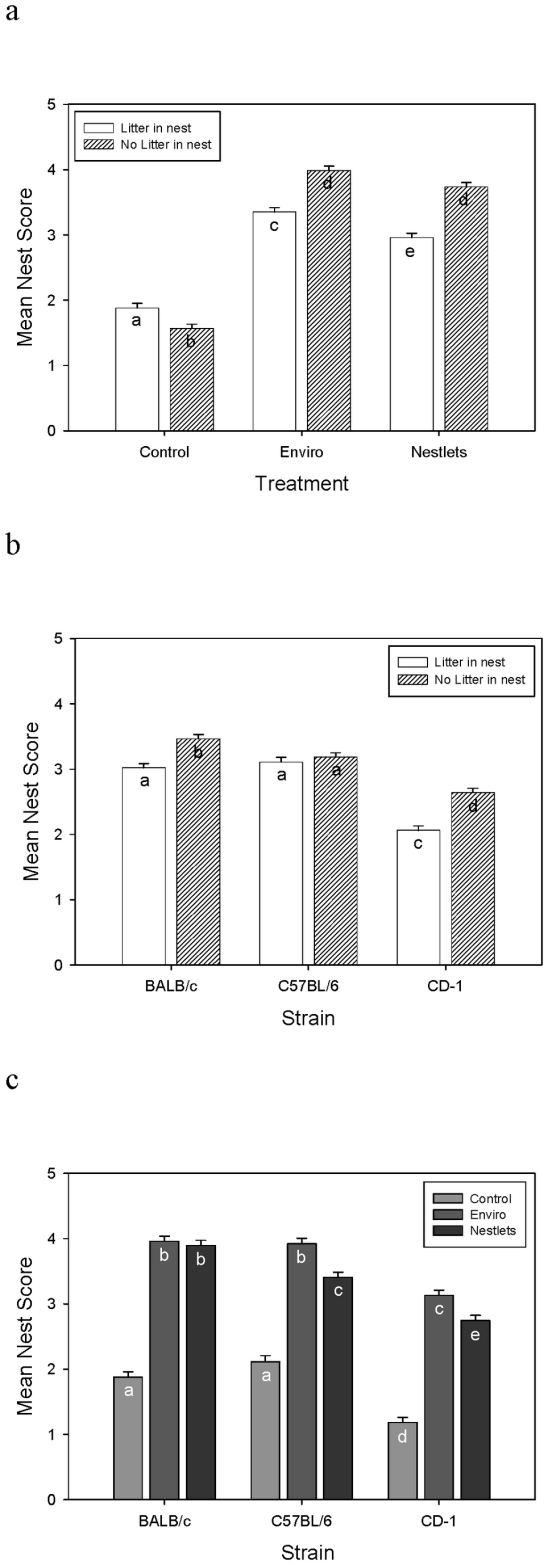
LSM and SE values of mean nest score for (a) Treatment by litter presence, (b) Strain by litter presence, and (c) Strain by treatment averaged by cage over six month experiment. Letters indicate significant differences using Tukey tests.

Nest score were significantly altered by the nesting material treatment as well as the presence of a litter in the nest (GLM: F_2,81_ = 38.5; P < 0.001; Figure 1a). In control mice, nest scores were significantly higher when a litter was present compared to when no litter was present (Tukey: P < 0.05). In contrast, the nests of mice provided with nesting material on average scored higher when there was no litter present compared to when a litter was present (Tukey: P < 0.05).

Strains differed in the effect of whether a litter was in the nest (GLM: F_2,81_ = 7.2; P = 0.001; Figure 1b). CD-1 and BALB/c mice both built better nests when no litter was present (Tukey: P < 0.05). However, C57BL/6 mice showed no difference in nest score based on litter presence (Tukey: P > 0.05). The extent to which nest scores differed between the nesting material conditions varied among mouse strains (GLM: F_4,81_ = 5.12; P = 0.001; Figure 1c). BALB/c and C57BL/6 mice built similar nests with Enviro-Dri (Tukey: P > 0.05), but they were significantly higher scoring nests than CD-1s (Tukey: P < 0.05). When given Nestlets, BALB/c mice built the best scoring nests between the three strains (Tukey: P < 0.05). C57BL/6 mice built the next best nests, which were significantly higher scoring than CD-1 mice (Tukey: P < 0.05). When given the control nesting condition, C57BL/6 and BALB/c mice built similar nests (Tukey: P > 0.05), which were again better than CD-1mice (Tukey: P < 0.05).

### Breeding Performance

The number of pups born alive per cage over the 6 month experiment differed significantly between nesting treatments (GLM: F_2,83_ = 4.36; P = 0.02). Mice receiving Enviro-Dri (51.1 ± 3.0) delivered more live pups than control mice (39.2 ± 3.0; Tukey: P < 0.05) but the number of pups born to mice that received Nestlets did not significantly differ from the other two conditions (48.1 ± 3.0; Tukey: P > 0.05). Genetic composition also affected the average number of pups born alive (GLM: F_2,85_ = 95.5; P < 0.001). As expected, outbred CD-1 mice had significantly more pups than the two inbred strains tested (79.0 ± 3.0; Tukey: P < 0.05) but the inbred strains (BALB/c 33.8 ± 3.0; C57BL/6 mice 25.7 ± 3.0) did not differ in the total number of pups born (Tukey: P > 0.05).

The number of pups weaned was significantly altered by the nesting treatment provided (GLM: F_2,85_ = 5.21; P = 0.007). Both nesting materials resulted in more pups weaned (Enviro-Dri: 49.1 ± 2.9; Nestlets: 46.7 ± 2.9) compared to controls (36.6 ± 2.9; Tukey: P < 0.05). Mouse strain also affected the average number of pups weaned (GLM: F_2,85_ = 102.5; P < 0.001). Outbred CD-1 mice weaned significantly more pups (77.5 ± 2.9) over the 6 month period than the two inbred strains (Tukey: P < 0.05). BALB/c mice weaned 32.9 ± 2.9 pups, significantly more than C57BL/6 mice (21.9 ± 2.9; Tukey: P < 0.05).

**Figure 2:**
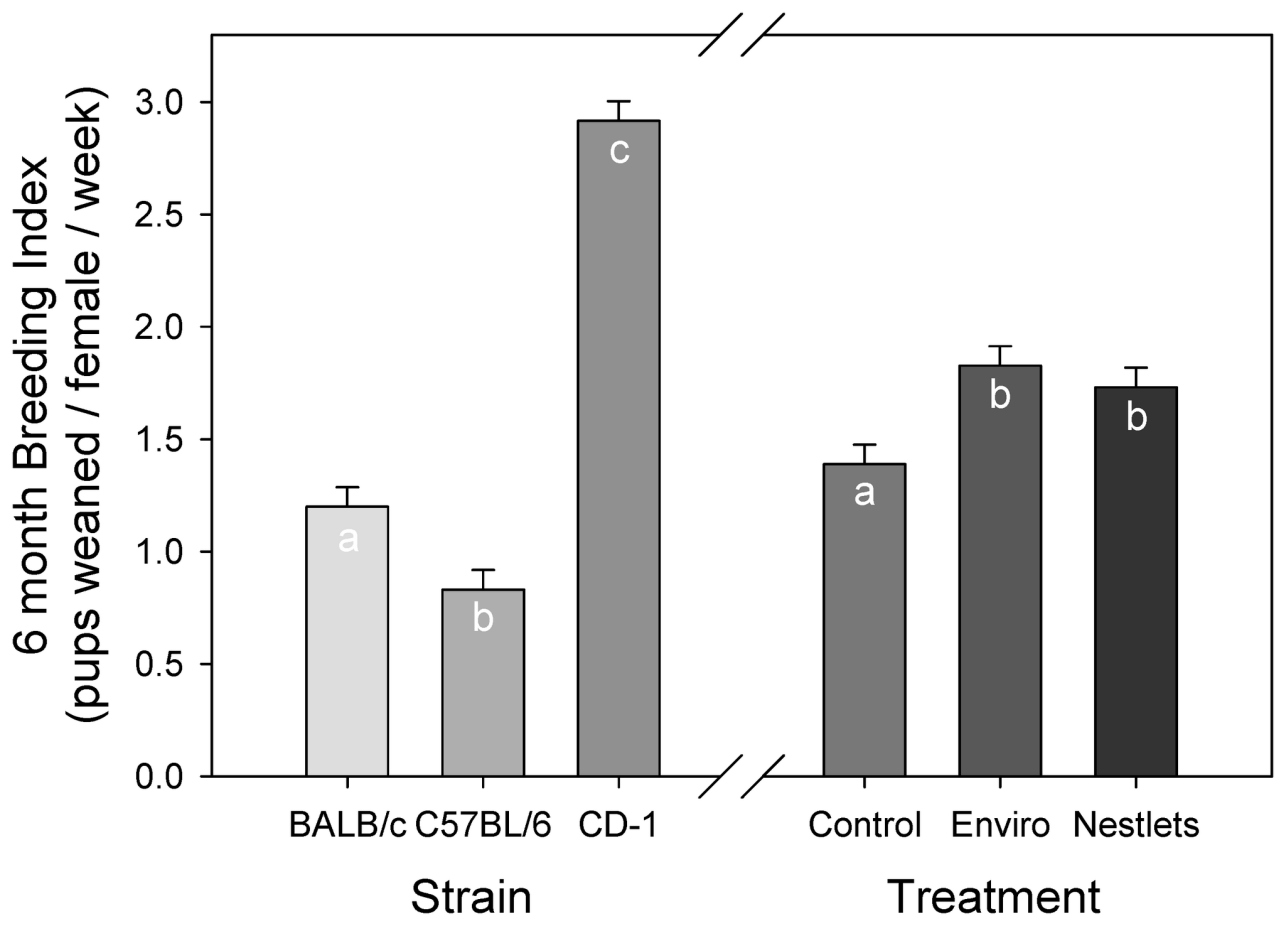
Mean six month breeding index is the number of pups weaned per female in a cage per week of pairing. LSM and SE values are plotted, and letters indicate significant differences using Tukey tests.

BI was also significantly improved by the provision of nesting material (GLM: F_2,85_ = 6.9; P = 0.002; Figure 2); mice that received Enviro-Dri (1.8 ± 0.08) or Nestlets (1.7 ± 0.08) had significantly higher breeding indices than controls (1.3 ± 0.08; Tukey: P < 0.05). Mouse strains significantly differed on their BI (GLM: F_2,85_ = 161.9; P < 0.001). As expected, the outbred CD-1 mice had the highest BI (2.91 ± 0.08), which was significantly different from the two inbred strains tested (Tukey: P < 0.05). BALB/c mice had a BI of 1.19 ± 0.08 which was significantly greater than C57BL/6 mice (0.83 ± 0.08; Tukey: P < 0.05).

**Figure 3:**
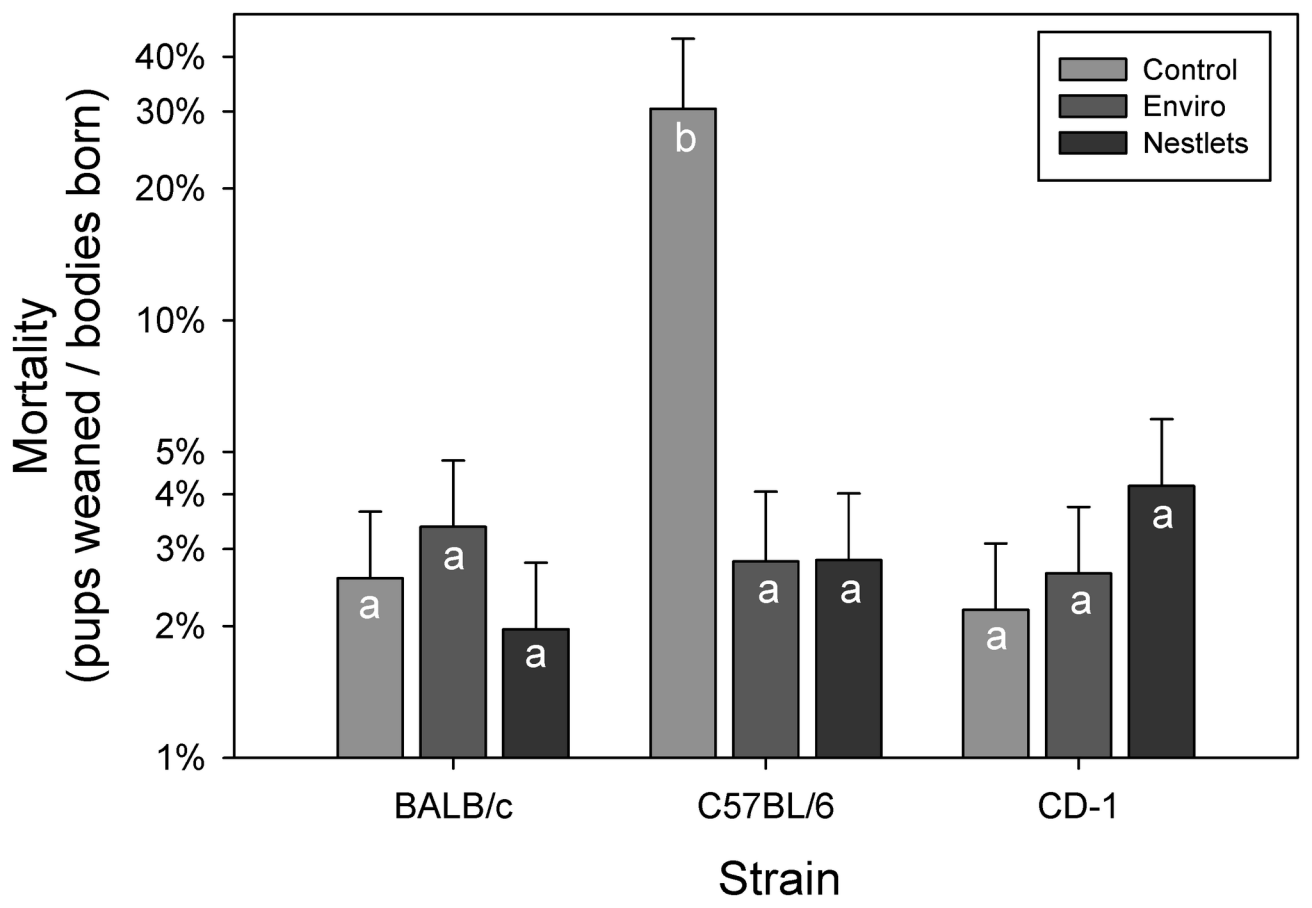
Mean pup mortality was calculated as the total number of bodies born by the total number of pups weaned. LSM and SE values are plotted, and letters indicate significant differences using Tukey tests.

An interaction between nesting treatment and strain significantly altered pup mortality (GLM: F_4,79_ = 6.38; P < 0.001; Figure 3). C57BL/6 mice were the only strain to show a significant reduction in percent mortality due to the nesting treatments (Tukey: P < 0.05). Control C57BL/6 mice had approximately 30% mortality, which was nearly thirteen times higher than the controls in the other two strains.

**Figure 4:**
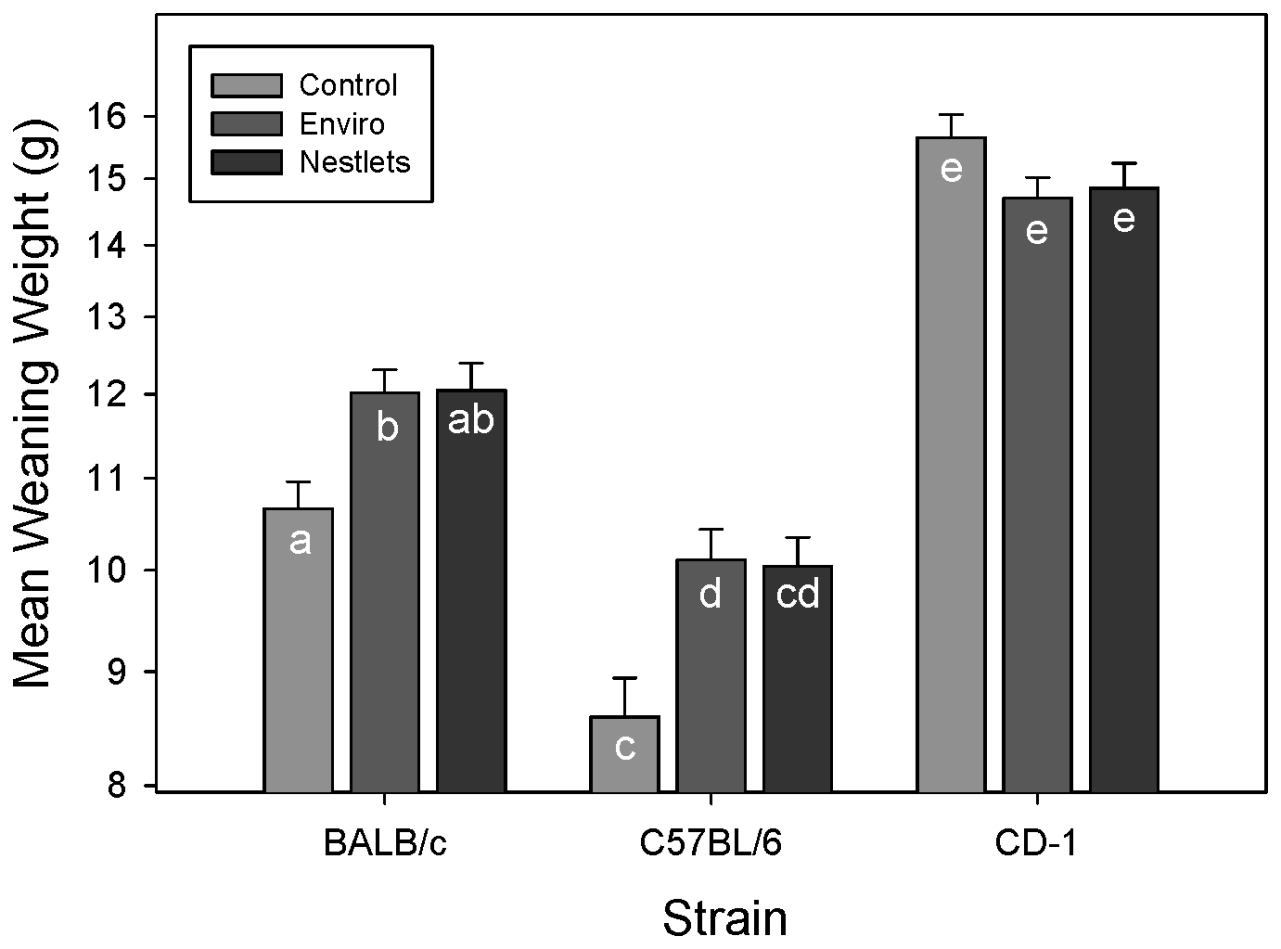
Mean weaning weight of pups raised in cages with different nesting material treatments is represented on the y axis as a log transformed scale. LSM and SE are plotted and significant differences using Tukey comparisons are indicated by asterisks.

**Figure 5:**
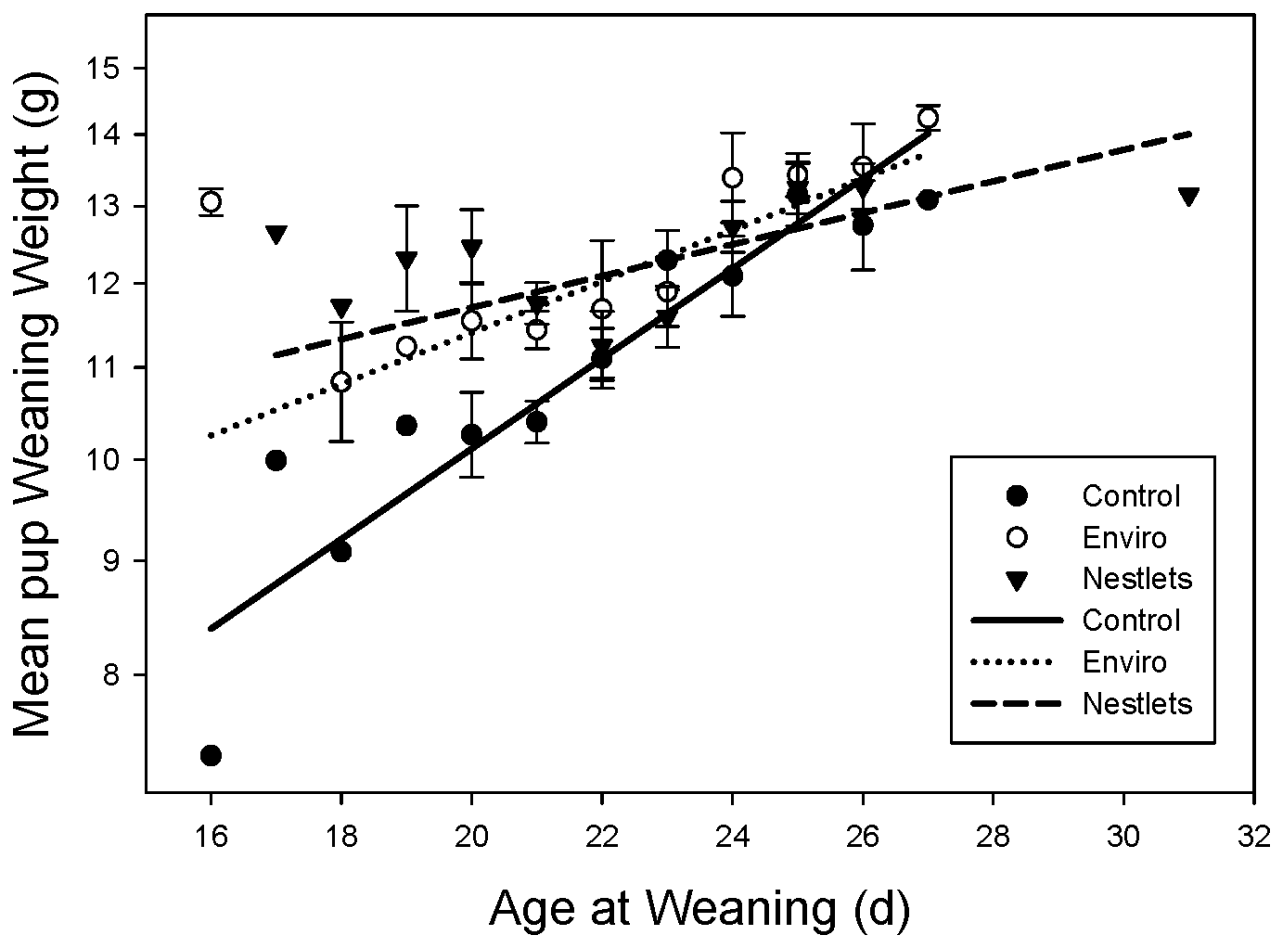
Mean pup weaning weight (g) is represented on the y axis as a log transformed scale. Observations at each weaning age were averaged per treatment for visual purposes. Data points without SE bars depict a singular observation at that weaning age. Solid and fragmented lines depict the least squares line for each treatment.

Nesting material treatments significantly altered the weaning weight of pups from inbred mice (GLM: F_4,318_ = 5.85; P < 0.001; Figure 4). BALB/c and C57BL/6 mice receiving Enviro-Dri weaned heavier pups than controls (Tukey: P < 0.05), while the weight of pups weaned from Nestlet cages did not differ significantly from either the control or Enviro-Dri (Tukey: P > 0.05). Nesting treatments in outbred CD-1 mice do not appear to affect pup weaning weights (Tukey: P > 0.05). The treatments also differentially affected weaning weight depending on the age at which the pups were weaned (GLM: F_2,318_ = 4.14; P = 0.02; Figure 5). The slope of weight gain for both nesting material treatments was significantly different from the controls (GLM: F_1,318_ = 7.25; P = 0.007). At 17 (Custom test: F_1,318_ = 16.03; P < 0.001) and 21 days of age (Custom test: F_1,318_ = 21.3; P < 0.001), mice from cages with nesting material were significantly heavier than control mice. However, if mice were weaned at 25 (Custom test: F_1,318_ = 0.041; P = 0.84) there was no difference in body weight.

### Food consumption

Nesting material conditions did not significantly affect the total amount of food consumed by mice over the 6 month breeding period (GLM: F_2,82_ = 2.82; P = 0.065). However, the three strains did show differences in food consumption (GLM: F_2,82_ = 19.8; P < 0.001). Outbred CD-1 mice ate significantly more food (4056.1 ± 105.9g) than did C57BL/6 (3091.4 ± 79.2g) and BALB/c mice (3122.4 ± 69.8g) over the 6 month experiment (Tukey: P < 0.05).

**Figure 6:**
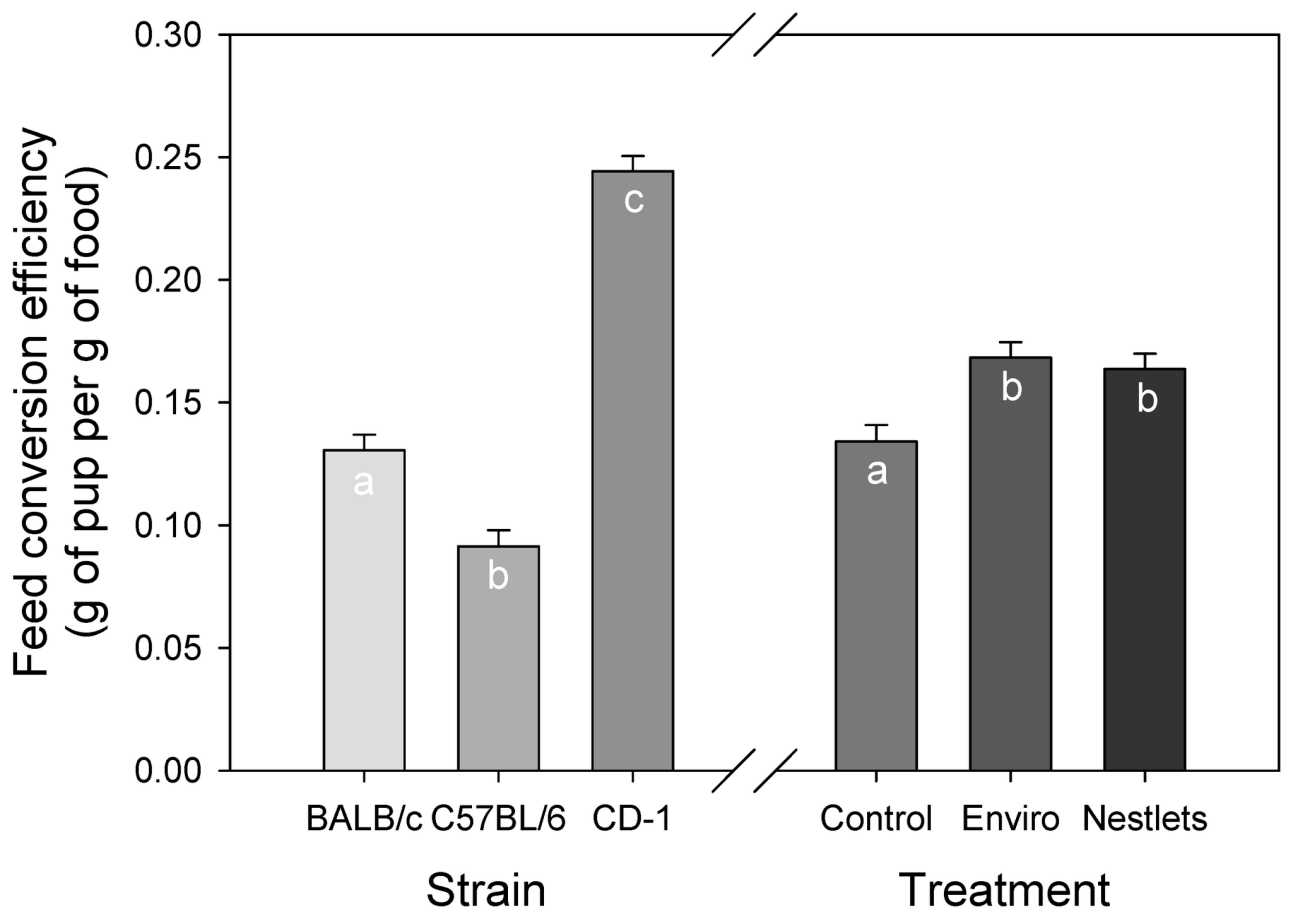
Mean number of pups weaned per kilogram of food. LSM values have been normalized to depict the number weaned per 1000 grams of food. Letters indicate significant differences using Tukey tests.

Although the amount of food eaten by mice in all the treatments was not significantly different, the nesting material treatments significantly altered the conversion of food to pups weaned (GLM: F_2,84_ = 6.0; P = 0.004; Figure 6). Enviro-Dri and Nestlets weaned approximately 12.5 ± 0.59 and 12.4 ± 0.59 pups respectively per kilogram of food. This was significantly more pups weaned than control mice (9.9 ± 0.61; Tukey: P < 0.05). Strains also differed in the number of pups weaned (GLM: F_2,84_ = 41.5; P < 0.001). On average, CD-1 mice weaned approximately 15.7 ± 0.61 pups per kilogram of food, which was significantly higher than BALB/c mice (11.1 ± 0.59) and C57BL/6 mice (8.1 ± 0.59; Tukey: P < 0.05).

## Discussion

The provision of 8g of nesting material (either Enviro-dri or Nestlets) significantly improved breeding performance in two commonly used inbred strains and one outbred stock. All three types of mice built higher scoring nests when provided either type of nesting material. Interestingly, nests scores differed both depending on the nesting treatment and whether there was a litter present in the nest at the time of nest scoring. Lactating mice have been reported to build superior nests compared to pregnant and virgin mice [21]. Levels of maternal progesterone have also been found to correlate with nest building [22,23] and the resulting improvements are thought to predict the survivability of the pups in the wild [15,16]. Our control animals displayed the same increase in nest score when pups were present in the nest. However, other research has documented decreases in nest building after parturition [24], as was seen in the mice receiving nesting material. The energetic demands of lactation render females hyperthermic, and the increased need for heat dissipation may be the reason for the decrease in nest score [25–27]. Others have proposed that as pups develop fur and their own endothermy, huddling and lower nest building are sufficient means of behavioral thermoregulation [24]. Our data provide a possible explanation for this apparent disagreement in the literature. Mice receiving either type of nesting material had decreased nest scores when pups were in the nest, while controls showed the opposite effect. This suggests that the overall improved nest quality provided by nesting material may have made nests too warm for females and therefore nests became more open to increase heat loss for the female; whereas control mice still needed to improve nest quality to optimize pup thermoregulation.

Nesting material may help the hyperthermic females and endothermically incompetent pups find some middle ground in terms of thermal comfort. On average, 11 more pups were weaned at heavier weights by BALB/c and C57BL/6 mice with Enviro-Dri. Parental investment is often partitioned between the number of offspring or the quality, or survivability, of those offspring [28]. Generally, increasing the number of offspring decreases their quality because the same parental resources must now be split between a larger group [28]. Since the inbred mice with Enviro-Dri produced more pups of improved quality, more parental resources must have been available to be dispersed amongst, and utilized by, the offspring for growth. This appears to be especially true during the earlier growth phase of the pups (less than 25 days of age). This idea is further evidenced by equal food consumption between the treatments over the 6 month period. Therefore, increased reproductive output cannot be from increased food consumption but from increased resource availability for pup growth and development.

Outbred CD-1 mice can be almost double the size of BALB/c mice at 7-8 weeks old. The combination of larger size and lactation-induced hyperthermia [25,27] may have affected CD-1 mice more than the smaller C57BL/6 and BALB/c mice. It is also possible that this result is confounded with room. Unfortunately, this confound could not be avoided since the main goal of this experiment was to determine the effect of providing two types of nesting material to mice in a production setting. C57BL/6 and BALB/c mice were housed in the same room but comparisons to CD-1 mice should be made cautiously, because we were unable to determine if the external environment or the animals themselves were driving the differences seen.

C57BL/6, unlike other mice used in this study, did not show any nest building differences when a litter was present in the nest. C57BL/6 mice are often considered poor nest builders [18], but this may have to do with thermal sensitivity as well as the material they are provided for nest building. C57BL/6 [18] as well as CD-1 mice (unpublished data) have shown difficulty with building using materials that are highly compressed. While the Nestlets used in this study appeared to be less compressed compared to similar compressed cotton nesting material used in other studies, these two strains built higher-scoring nests with Enviro-Dri. BALB/c mice however, do not appear to have trouble processing compressed materials (unpublished data).

When provided with enough nesting material, pup mortality in C57BL/6 mice was reduced by nearly 27%. Previous research suggests that this strain favors the thermoregulatory behavior of thermotaxis (movement in response to temperature) over nest building [5]. Nest building of C57BL/6 mice does respond to temperature, where mice build more enclosed nests in cooler temperatures [5]. Regardless of their primary mode of behavioral thermoregulation, providing materials C57BL/6 are better able to build with appears to improve survivability of pups, likely through improved insulation.

Breeding performance is also easily influenced by stressors other than the thermal stress explored in this paper [29]. The absence of a retreat or hiding place in various species has been shown to be stressful [30], resulting in stereotypy development and heightened behavioral indicators of fearfulness [31–33]. The psychological benefits of providing mice with a retreat space may also improve overall well-being and may have also impacted breeding performance. Thus, the provision of materials to produce a naturalistic retreat space or nest may be an avenue to decreasing stress associated with various aversive stimuli [30].

Nesting material provisions in laboratory mice produce two major benefits to end-users. First, a reduction of thermal stress renders mice better models for experimentation. Animals housed at or just below recommended housing temperatures are under thermal stress and have altered metabolism, behavior, and immune function [1,4,34]. Increased metabolism, in turn, can induce a variety of diseases via increased generation of oxidative stress during normal metabolism [35]; these profound and wide reaching changes can easily alter scientific outcomes. For instance immune-suppressed animals may not respond to vaccination [36] or animals with elevated metabolic rates may have altered pharmacokinetics [37].

The second benefit is the mean increase in breeding performance. An increase in 0.5 and 0.4 pups per week from Enviro-Dri and Nestlets respectively, results in a substantial monetary gain, especially for poor breeding strains. A single C57BL/6 mouse, the most widely used inbred strain, costs between $16–26, depending on the weight and age [38]. This small increase yielded 13 more weanlings on average, grossing an extra $273 per cage at the average price ($21). In addition to the increased productivity per cage, less food is needed to produce 1 pup. Thus, nesting material results in more pups produced at a lower cost. Considering the increase in revenue from each cage, an increased cost of $0.624 for Enviro-Dri or $3.24 for Nestlets per cage over the 6 month breeding period is a sensible expenditure.

We conclude that nesting material, via increased insulation, frees up energetic resources from thermogenesis which were reallocated to improve reproductive performance. All strains built higher scoring nests when given either Enviro-Dri or Nestlets. This resulted in more pups born, and weaned, and a decrease in pup mortality in C57BL/6 mice. In addition, the amount of food needed to produce one pup was significantly decreased when cages were provided with nesting material. Improved nest building most likely improves the thermal microenvironment for both adults as well as pups. The addition of nesting material is both a cost effective and simple enrichment that can be provided to the home cage to eliminate thermal stress as well as improve reproductive performance.
